# Enhancement of PCR Sensitivity and Yield Using Thiol-modified Primers

**DOI:** 10.1038/s41598-018-33223-2

**Published:** 2018-10-05

**Authors:** Yalong Bai, Yi Xiao, Yujuan Suo, Yuanyuan Shen, Yi Shao, Donglai Zhang, Changyan Zhou

**Affiliations:** 1 Institute for Agro-food Standards and Testing Technology, Laboratory of Quality & Safety Risk Assessment for Agro-products (Shanghai), Ministry of Agriculture, Shanghai Academy of Agricultural Sciences, Shanghai, 201403 China; 2 Department of Chemistry and Biochemistry, Florida International University, 11200 SW Eighth Street, Miami, Florida 33199 United States

## Abstract

Various additives can enhance the quality of PCR amplification, but these generally require considerable optimization to achieve peak performance. Here, we demonstrate that the use of thiol-modified primers can enhance both PCR sensitivity and yield. In experiments with *V*. *parahaemolyticus* genomic DNA, this primer modification enhances PCR sensitivity by more than 100-fold, with accompanying improvements in amplicon yield. Then, an artificial plasmid with the same primer binding regions and different internal amplification sequence was designed. The result showed that the amplification also be improved by using the same thiol-modified primers. It indicated the enhancement was not caused by the effect of the thiol-modified primers on the second structure of amplification sequence. Subsequent experiments demonstrate that the effects of this modification are potentially due to altered interaction between the primers and proteins in the reaction mixture. Amplification with thiol-modified primers was strongly inhibited by the presence of extraneous proteins relative to standard DNA primers, which indicates that thiol-modified primers may be inhibited due to interaction with these proteins. In contaminant-free reactions, however, the thiol-modified primers might interact more strongly with DNA polymerase, which could in turn improve PCR amplification.

## Introduction

The polymerase chain reaction (PCR) has become a core molecular biology technique due to its powerful capability to amplify large quantities of specific segments of DNA from very limited amounts of template *in vitro*^1,2^. PCR is now being applied in a wide range of experimental contexts, including genome sequencing, genetic engineering, mutation detection, analysis of gene expression, diagnosis of genetic diseases, forensic analysis of biological evidence, and detection of bacterial and viral pathogens^3,4^.

PCR amplification cannot occur robustly in certain experimental conditions, and there are now a large number of modifications to the basic PCR assay designed to enhance its specificity, efficiency, yield and fidelity. One of these entails the use of specialized DNA polymerases, such as hot-start and high-fidelity DNA polymerase. For PCR reactions using hot-start DNA polymerase, amplification does not begin until the reaction reaches the annealing temperature. This helps to prevent primer/template mispriming and the formation of primer dimers, leading to greatly improved specificity and sensitivity^5^.

One can also employ a variety of PCR additives, including organic or biological reagents and nanomaterials. Some of the organic and biological reagents used as additives include formamide^6^, dimethyl sulfoxide (DMSO)^7^, tetramethylammonium chloride^8^, and single-stranded binding protein^1^. More recently, researchers have identified numerous nanomaterials that can enhance the yield of PCR, such as silver nanoparticles^9^, gold nanoparticles^9,10^, magnetic nanoparticles^9^, TiO_2_ nanoparticles^10^, carbon-coated silica nanoparticles^11^, and graphene-ZnO nanocomposites^12^. Nanomaterials can also improve the specificity of PCR, including gold nanoparticles^13^, poly(diallyldimethylammonium chloride)-protected gold nanoparticles^14^, dendrimers^15^, amino-modified magnetic nanoparticles^16^, and graphene oxide^17^. Furthermore, some researchers have also identified nanomaterials that can improve the efficiency^18^ and fidelity^19^ of PCR.

However, the amount of the additives must be optimized for different PCR systems, as low concentrations of an additive may not improve the amplification of PCR whereas excessive additive could inhibit amplification^9^. As such, considerable optimization is required to identify the strategy best suited for amplification under different PCR conditions.

In this study, we have replaced standard DNA primers with thiol-modified primers to complete PCR. Our results indicated that this modification enhanced both the sensitivity and yield of PCR. This strategy dese not need any optimization steps. To our knowledge, it was first proposal that the modification of primers were used to optimize PCR.

## Results

### Thiol-modified primers improve PCR sensitivity in an assay for *V*. *parahaemolyticus* genomic DNA

**Figure 1:**
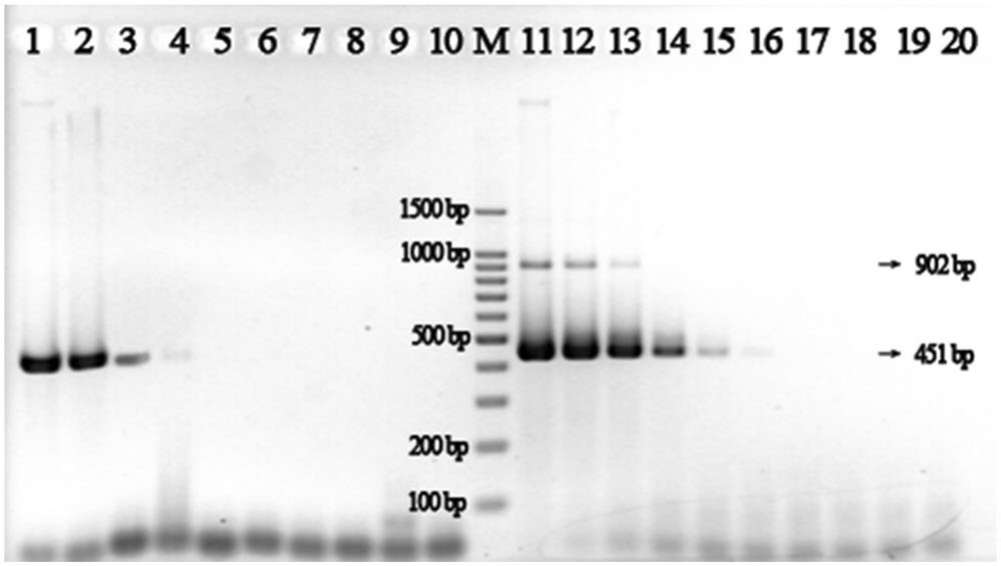
Sensitivity of a PCR assay for *Vibrio parahaemolyticus* genomic DNA using standard primers V451-r/f (lanes 1–9) and thiol-modified primers V451S-r/f (lanes 11–19). Lane M: Marker; Lanes 1–9 & 11–19: reactions containing the following amounts of their respective template: 50 ng, 5 ng, 500 pg, 50 pg, 5 pg, 500 fg, 50 fg, 5 fg, 0.5 fg. Lane 10 & 20: negative control without DNA template.

*Vibrio parahaemolyticus* is a Gram-negative, halophilic pathogen that is sometimes present in seafood, and produces an infection that results in symptoms including fever, headache, diarrhea, nausea, and chills^20^. In this study, *V*. *parahaemolyticus* (CMCC50115, purchased from HuanKai Bio Inc., Guangdong, China China) was gown overnight in Luria-Bertani (LB) broth with 3% NaCl at 37 °C under agitation. One milliliter of broth was used to isolate *V*. *parahaemolyticus* genomic DNA by EZ-10 Spin Column Bacterial Genomic DNA Isolation Kit (Sangon, Shanghai, China) according to manufacturer’s instructions. The resulting DNA solution (50 ng/μL) was diluted 10-fold dilutions from 0.5 fg/μL to 50 ng/μL using sterile water. We used these diluted solutions as templates for two sets of PCR assays, one set using standard DNA primers, and the other using thiol-modified primers (Fig. 1). We found that the sensitivity of PCR assays using thiol-modified primers (V451S-f/r) was at least 100 times higher than the reactions using standard primers (V451-f/r). For the V451S-f/r reaction, we detected long amplification products of ~900 base-pairs (bp) along with the predicted target products (451 bp). We predict that these new products arise from the linkage of two copies of target product by the formation of disulfide bonds, which would have a predicted length of 902 bp.

**Figure 2:**
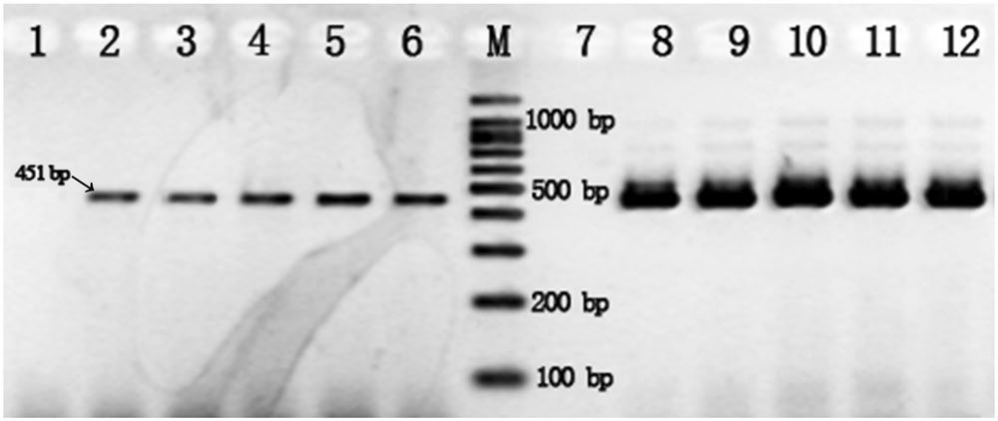
The yield of PCR amplification for *V*. *parahaemolyticus* genomic DNA using standard primers V451-r/f (lanes 2–6) and thiol-modified primers V451S-r/f (lanes 8–12). Lanes 2–6 & 8–12: reactions with 500 pg of *V*. *parahaemolyticus* genomic DNA. Lanes 1 & 7: negative control without DNA template.

We also found that the use of thiol-modified primers improved the yield of PCR amplification relative to standard primers. We performed another pair of PCR reactions with 2 μL of 500 pg/μL *V*. *parahaemolyticus* genomic DNA as a template (Fig. 2), and found that the yield with thiol-modified primers was about 5.3 times higher than the group using standard primers, which was calculated by Image Lab 6.0 (Bio-Rad, Hercules, CA, USA).

### Exploration of the relationship between secondary structure of the amplification sequences and thiol-modified primers

**Figure 3:**
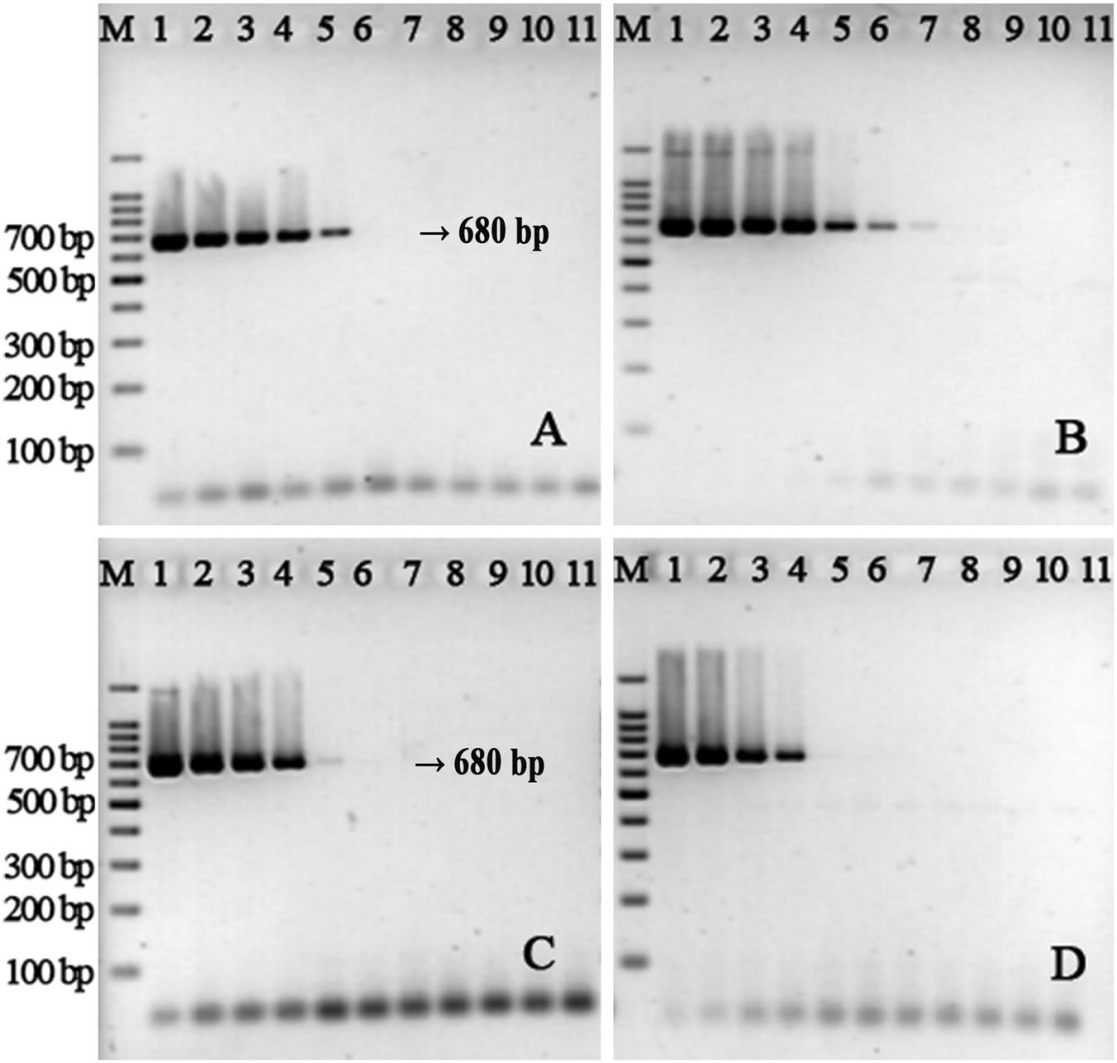
PCR amplification of artificial plasmids using V451-r/f (**A**), V451S-r/f (**B**), V451-r & V451S-f (**C**) and V451S-r & V451-f (**D**). Lane M: Marker; Lanes 1–10: reactions with 190 pg, 19 pg, 1.9 pg, 190 fg, 19 fg, 1.9 fg, 190 ag, 19 ag, 1.9 ag, and 0.19 ag of plasmid DNA template. Lane 11: negative control without DNA template.

Secondary structures in the DNA template can sometimes hinder primer extension by DNA polymerase, leading to reduced amplification^21^. We wondered whether thiol-modified primers promoted PCR amplification because of their effect on the complex secondary structures. To test this hypothesis, we constructed an artificial plasmid which contained the same primer binding regions for V451-r/f or V451S-r/f, but with completely different sequences between the primer-binding regions relative to the equivalent sequences from the *V*. *parahaemolyticus* genome, based on data obtained via BLAST analysis. The resulting artificial plasmids could be amplified using V451-f/r or V451S-f/r, but be of entirely different secondary structure in the region between the two primer-binding regions. We used a serial dilution of these plasmids at concentrations ranging from 0.19 ag/μL to 190 pg/μL as templates for PCR assays with both standard and thiol-modified primers (Fig. 3A,B). We determined that the sensitivity of detection was again enhanced by using the thiol-modified primers, which indicates that the improved PCR performance arising from the use of thiol-modified primers is not primarily due to their effects on the secondary structure. We also investigated the extent of PCR amplification enhancement when using a thiol-modified primer and a standard primer (V451-r & V451S-f and V451S-r & V451-f) on the artificial plasmid template, and found that the sensitivity was not enhanced when only one of the two primers was thiol-modified (Fig. 3C,D).

### The mechanism of the enhancement of PCR amplification caused by thiol-modified primers

**Figure 4:**
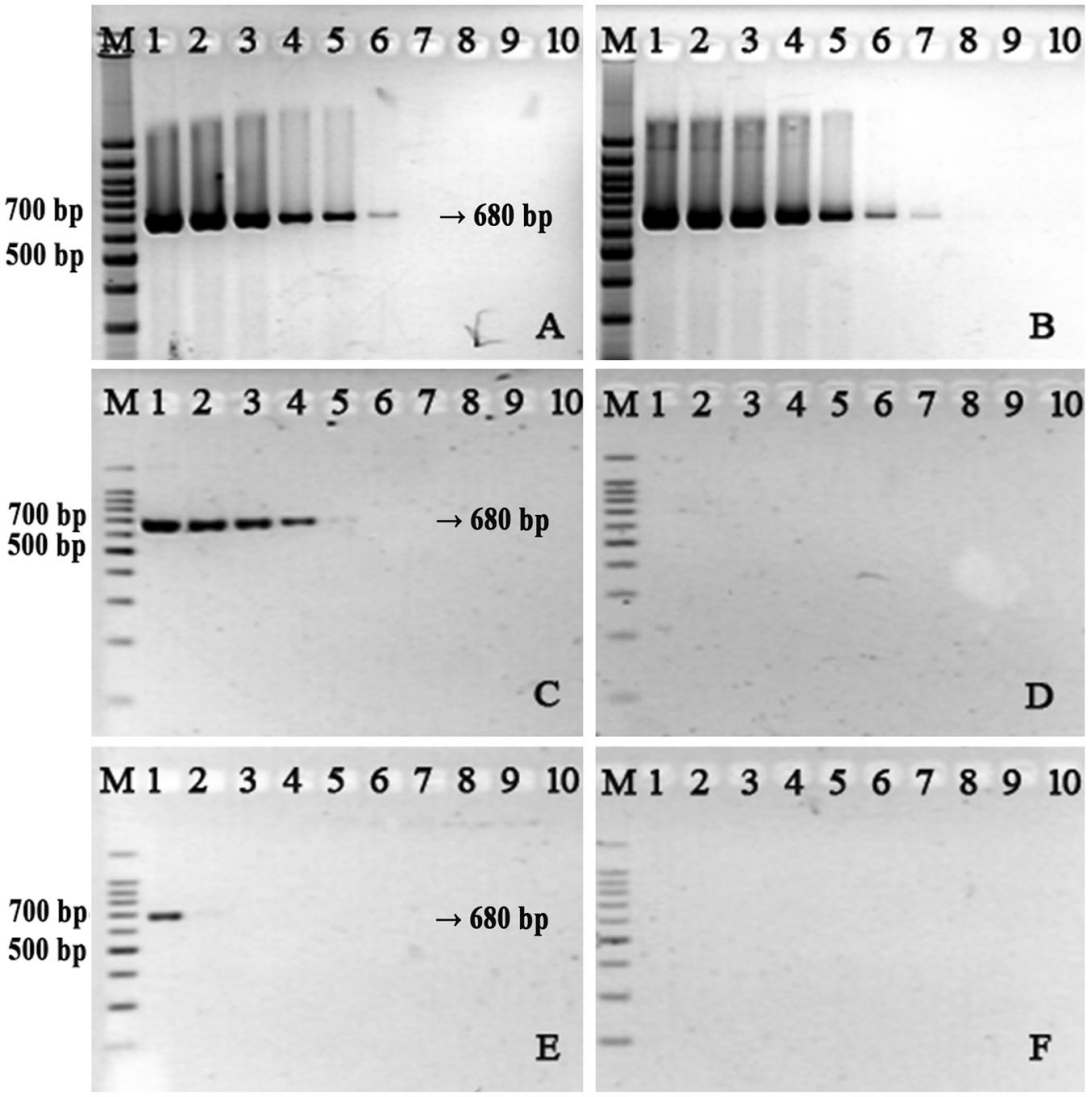
PCR amplification of artificial plasmids using standard V451-r/f primers (**A**,**C**,**E**) and thiol-modified V451S-r/f primers (**B**,**D**,**F**) in the presence of LB broth (**A**,**B**: 1 μL; **C**,**D**: 2 μL; **E**,**F**: 4 μL). Lane M: Marker. Lanes 1–9: reactions with 190 pg, 19 pg, 1.9 pg, 190 fg, 19 fg, 1.9 fg, 190 ag, 19 ag, and 1.9 ag of DNA template; Lane 10: negative control without DNA template.

PCR is generally sensitive to foreign materials, which tend to inhibit amplification. We therefore examined the extent to which interferents in the reaction inhibit PCR efficiency using standard primers versus thiol-modified primers. We used LB broth as a model, because residual bacterial broth in DNA template preparations is known to inhibit PCR amplification^22^. Our results showed that reactions using thiol-modified primers were much more sensitive to LB broth than those using standard primers (Fig. 4). When we added 2 μL of LB, the amplification products were completely inhibited in reactions with thiol-modified primers, whereas successful amplification occurred in reactions containing ≥19 fg of DNA templates with standard primers; indeed, amplification products could be detected with standard primers even when 4 μL of broth was added. These results indicate that PCR amplification with thiol-modified primers is extremely sensitive to LB broth. The main components of this broth are tryptone and yeast extract. Thereby, we hypothesized that the interaction between the thiol-modified primers and proteins might be the reason that the amplifications were inhibited.

**Figure 5:**
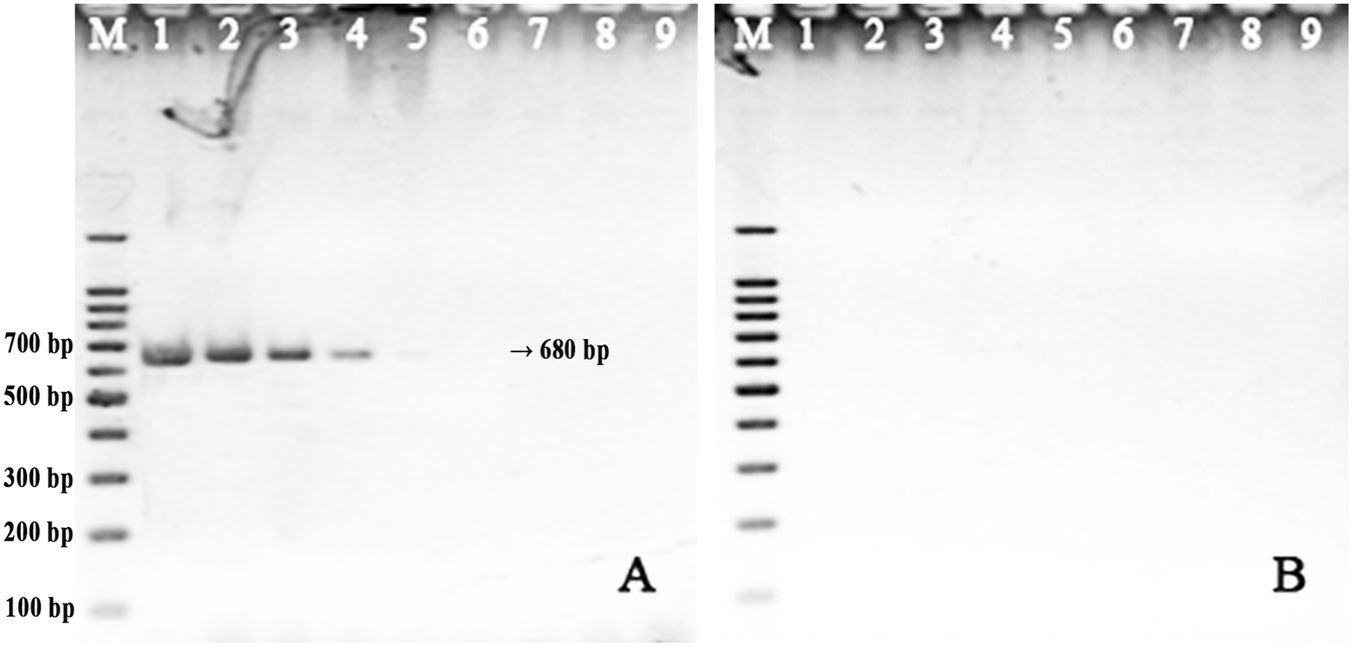
PCR amplification of artificial plasmids using standard V451-r/f primers (**A**) and thiol-modified V451S-r/f primers (**B**) with 2 μL of 10% BSA. Lane M: Marker; Lanes 1–8: reactions with 190 pg, 19 pg, 1.9 pg, 190 fg, 19 fg, 1.9 fg, 190 ag, and 19 ag of DNA template. Lane 9: negative control without DNA template.

To test whether contaminating protein has an especially pronounced inhibitory effect on thiol-modified primers, we performed another set of PCR reactions with 2 μL of 10% bovine serum albumin (BSA). As above, the reactions using thiol-modified primers were inhibited completely by the presence of this contaminating protein, while the reactions using standard primers exhibited only a modest decrease in sensitivity (Fig. 5). One possibility is that the thiol group facilitates primer interaction with proteins in general essentially acting as a double-edged sword. On one hand, thiol-modified primers might tend to interact with DNA polymerase protein more strongly than standard DNA primers, leading to enhancement of PCR sensitivity and yield. However, the presence of contaminating protein would result in a sharp decrease in amplification efficiency.

Finally, we used amino- and carboxyl-modified versions of the same primer pairs in order to compare amplification efficiency relative to standard primers. Neither primer pair exhibited any notable improvement or reduction in amplification efficiency relative to unmodified primers (data not shown), indicating that primers modified with different electric charges do not have a meaningful effect on PCR relative to the improved performance seen with thiol-modified sequences.

**Figure 6:**
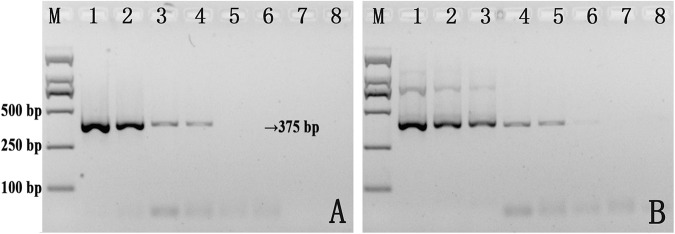
Sensitivity of a PCR assay for *Salmonella* genomic DNA using standard Sa375-r/f primers (**A**) and thiol-modified Sa375S-r/f primers (**B**). Lane M: Marker. Lanes 1–7: reactions with 2 μL of 70 ng/μL, 7 ng/μL, 700 pg/μL, 70 pg/μL, 7 pg/μL, 700 fg/μL, 70 fg/μL DNA template. Lane 8: negative control without DNA template.

We further determined that thiol-modified primers are also effective for bolstering the efficiency of PCR reactions for which sensitivity is low when standard primer pairs were used. For example, we observed a detection limit of 140 pg for *Salmonella* genomic DNA (CMCC50115, purchased from HuanKai Bio Inc., Guangdong, China), whereas the detection limit with thiol-modified primer pairs Sa375S-r/f was two orders of magnitude lower, at 1.4 pg (Fig. 6). This is similar to our results in the reactions using the standard V451-r/f DNA primers to detect *V*. *parahaemolyticus* genomic DNA, where the detection limit was 50 pg, relative to 500 fg when using thiol-modified primer pairs. However, in assays where the detection limit using standard primer pairs for bacterial genetic DNA was already very low on the order of 0.1–1.0 pg (≈22.8–228 copies/PCR^23^), we found that amplification could not be further improved with thiol-modified primers (data not shown). This may be because the amplification reactions had reached an inherent limit of sensitivity. Moreover, we also analyzed the amplification length and GC content of a large number of DNA templates, and we did not find any relationship between these factors and enhancement of PCR (data not shown).

## Discussion

Many modifications based on basic PCR to enhance the quality of amplifications except sophisticated optimizations were reported previously, but most of these entail the use of an additive in the reactions. In the present study, we describe the use of thiol-modified primers to enhance PCR sensitivity and yield. To our knowledge, it was the first time to improve PCR amplification based on primers modification. This improvement was simply a product of minor modification to the primers, and required no further optimization as is typically necessary for PCR that has been enhanced through the use of additives.

We explored why PCR amplification using thiol-modified primers could lead to enhancements of sensitivity and yield. Secondary structures present in the template can impact the sensitivity of PCR amplification usually, and we therefore generated an artificial plasmid with the same primer binding regions but a different internal amplification sequence. We determined that amplification exhibits similar improvement with thiol-modified primers for this template, even though the secondary structure of the sequence was completely different. This indicates that the enhancement in amplification was not due to the effect of the thiol-modified primers on template secondary structure. We also determined that both primers must be thiol-modified for amplification enhancement to occur.

However, we also determined that amplification with thiol-modified primers is extremely sensitive to the presence of contaminating proteins. This indicates that thiol-modified primers may be more prone to interacting with proteins. If these are extraneous proteins present in the sample, the amplification will be inhibited, whereas in the absence of interferent proteins, the thiol-modified primers would theoretically interact strongly with DNA polymerase. This could potentially explain why thiol-modified primers produce improved PCR amplification.

## Methods

### PCR amplification

**Figure 7:**
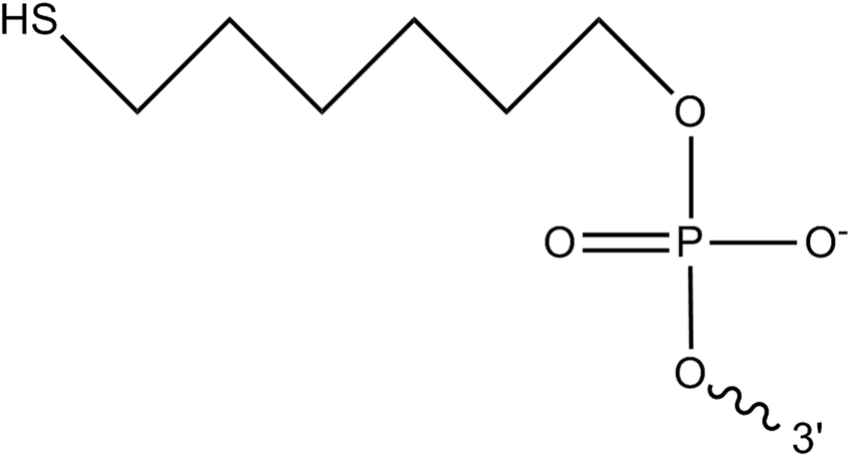
Schematic diagram of thiol-modified primer.

PCR was carried out in a 25-μL volume containing 75 mM Tris-HCl, 20 mM (NH_4_)_2_SO_4_, 1.5 mM MgCl_2_, 0.1 μM each dNTP, 0.2 μM each primer, 0.01% (v/v) Tween 20, 1 U of Taq polymerase (Thermo Fisher Scientific, Fremont, CA, USA), and a certain amount of DNA templates using a T100 thermal cycler (Bio-Rad, Hercules, CA, USA). All primers were designed using the Primer 5.0 program (Premier Biosoft International, Palo Alto, CA.) and were synthesized by Shanghai Sangon Corporation (Shanghai, China). The schematic diagram of thiol-modification primers is shown in Fig. 7. The resulting products were purified by using high performance liquid chromatography and capillary electrophoresis to eliminate impurities. All primer sequences are presented in Table 1.

**Table 1:** Primers for PCR amplifications.

Name	Sequences (5′-3′)	DNA templates sources	Amplicon length
V451-f	GGTGTCTTTCCAATCCTTTC	*Vibrio parahaemolyticus/*artificial plasmid	451 bp
V451-r	GGTATCGAACAACTGTAGCG		
V451S-f	HS-(CH_2_)_6_GGTGTCTTTCCAATCCTTTC	*Vibrio parahaemolyticus/*artificial plasmid	451 bp
V451S-r	HS-(CH_2_)_6_GGTATCGAACAACTGTAGCG		
LP683-f	GGTGTCTTTCCAATCCTTTCACAACCAGCAGGTCAAGG	Cottonseed	680 bp
LP683-r	GGTATCGAACAACTGTAGCGCCAGGGTCGCGTTATGAG		
C643-f	ACAACCAGCAGGTCAAGG	Cottonseed	640 bp
C643-r	CCAGGGTCGCGTTATGAG		
Sa375-f	GGAACCATCACGGACAATAC	*Salmonella*	375 bp
Sa375-r	GCCATCGGCAAGATAACG		
Sa375S-f	HS-(CH_2_)_6_GGAACCATCACGGACAATAC	*Salmonella*	375 bp
Sa375S-r	HS-(CH_2_)_6_GCCATCGGCAAGATAACG		

PCR reactions were performed with the following program: 94 °C for 5 min; 35 cycles of 94 °C for 30 s, 56 °C for 30 s, and 72 °C for 42 s; 72 °C for 8 min. The PCR products were analyzed by agarose gel electrophoresis with 4 S Red Plus Nucleic Acid Stain (TransGen Biotech, Beijing, China) and visualized using a Bio-Rad Gel Doc XR + Imaging System (Bio-Rad, Hercules, CA, USA).

### Construction of the artificial plasmid with the same primers binding regions

**Figure 8:**
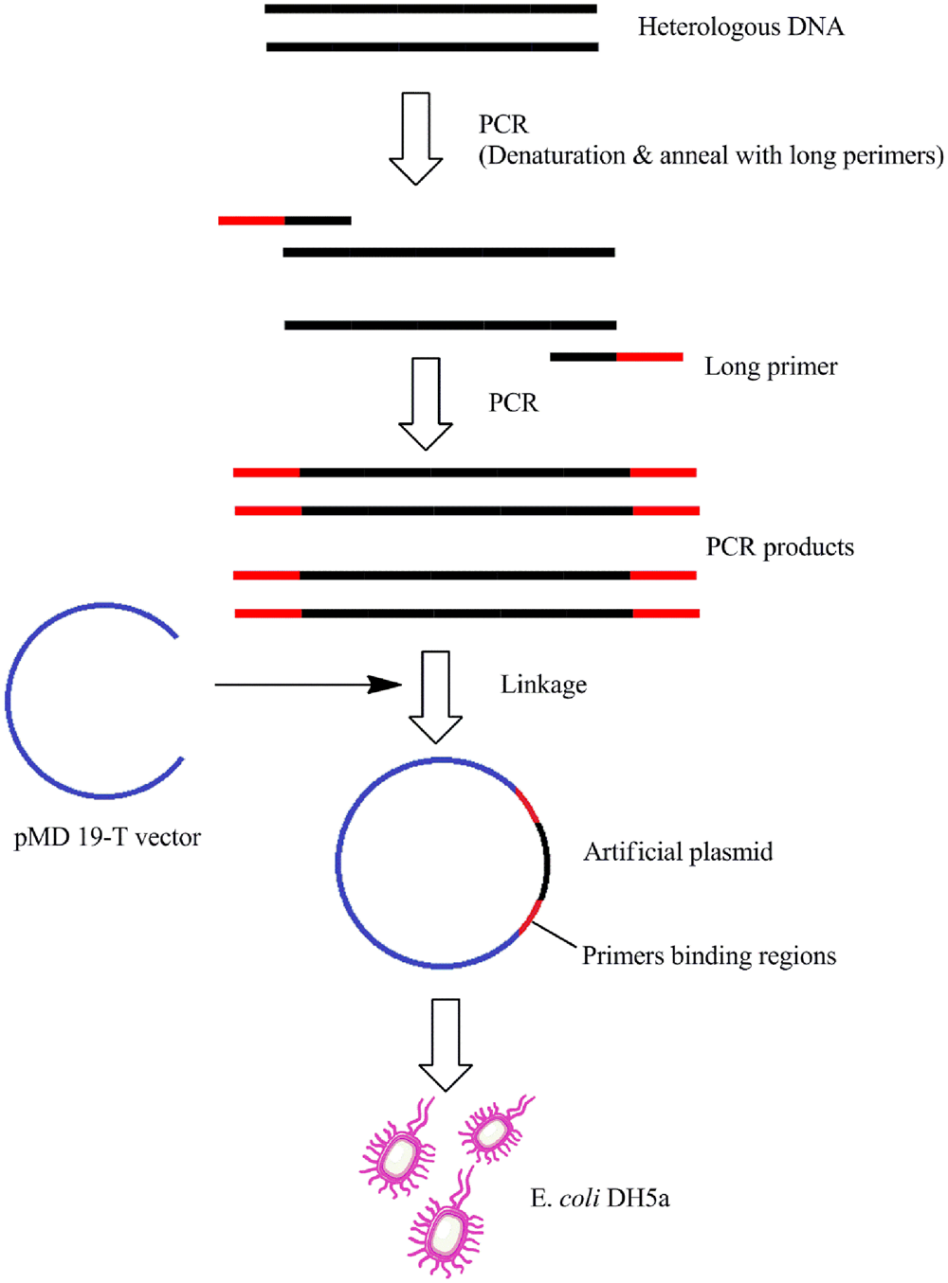
Schematic diagram of the procedure for constructing an artificial plasmid with the same primer-binding regions.

Synthetic amplification sequences were designed comprising the primer-binding regions from *V*. *parahaemolyticus* based on heterologous sequence derived from cotton genomic DNA. A pair of long oligonucleotide primers were used as a tool to bring in the primers binding regions referring to the composite primer technique^24^. The 5′ ends of the long primers (LP683-f/r) were identical to the sequences of the *V*. *parahaemolyticus* primers (V451-r/f), whereas the 3′ ends of the long primers were complementary to the cotton-derived sequence. The resulting amplification products carried the primer-binding regions at either end of the previous amplicons produced by the primers C643-f/r. These amplification products were ligated into pMD 19-T vectors (Takara, Dalian, China), and then transfected into *E*. *coli* DH5α. The schematic diagram of the whole process is shown in Fig. 8. After the cells containing the plasmids were cultivated overnight, the plasmid DNA was extracted by alkaline lysis^25^.

## References

[1] Rapley R (1994). Enhancing PCR amplification and sequencing using DNA-binding proteins. Mol. Biotechnol..

[2] Bai Y (2013). A rapid method for the detection of foodborne pathogens by extraction of a trace amount of DNA from raw milk based on amino-modified silica-coated magnetic nanoparticles and polymerase chain reaction. Anal. Chim. Acta..

[3] Erlich, H. *PCR technology: principles and applications for DNA amplificatio*n. Freeman & Co. 39–99 (2015).

[4] Bai Y (2016). Synthesis of amino-rich silica-coated magnetic nanoparticles for the efficient capture of DNA for PCR. Colloids Surf. B..

[5] Mi L (2009). Modulation of DNA Polymerases with Gold Nanoparticles and their Applications in Hot-Start PCR. Small.

[6] Sarkar G, Kapelner S, Sommer SS (1990). Formamide can dramatically improve the specificity of PCR. Nucleic Acids Res..

[7] Musso M, Bocciardi R, Parodi S, Ravazzolo R, Ceccherini I (2006). Betaine, Dimethyl Sulfoxide, and 7-Deaza-dGTP, a Powerful Mixture for Amplification of GC-Rich DNA Sequences. J. Mol. Diagn..

[8] Chevet E, Lemaître G, Katinka MD (1995). Low concentrations of tetramethylammonium chloride increase yield and specificity of PCR. Nucleic Acids Res..

[9] Kambli P, Kelkar-Mane V (2016). Nanosized Fe_3_O_4_ an efficient PCR yield enhancer—Comparative study with Au, Ag nanoparticles. Colloids Surf. B..

[10] Dantas PVP, Melo CF, Houllou LM, Machado G (2017). Nanoparticle-assisted Polymerase Chain Reaction (NanoPCR): Optimization of PCR detection of Leifsonia xyli subsp. xyli by the addition of nanoparticles. J. Adv. Res..

[11] Park JY (2015). Dopamine-Assisted Synthesis of Carbon-Coated Silica for PCR Enhancement. ACS Appl. Mater. Inter..

[12] Sharma V (2016). Studying the effect of Graphene-ZnO nanocomposites on Polymerase Chain Reaction. ACS Appl. Mater. Inter..

[13] Li H (2005). Nanoparticle PCR: nanogold-assisted PCR with enhanced specificity. Angew. Chem. Int. Ed. Engl..

[14] Yuan L, He Y (2013). Effect of surface charge of PDDA-protected gold nanoparticles on the specificity and efficiency of DNA polymerase chain reaction. Analyst.

[15] Cao X (2009). Enhanced specificity and efficiency of polymerase chain reactions using poly (amidoamine) dendrimers and derivatives. Analyst.

[16] Bai Y (2015). Nanoparticles Affect PCR Primarily via Surface Interactions with PCR Components: Using Amino-Modified Silica-Coated Magnetic Nanoparticles as a Main Model. ACS Appl. Mater. Inter..

[17] Zhong Y (2016). Enhancing the specificity of polymerase chain reaction by graphene oxide through surface modification: zwitterionic polymer is superior to other polymers with different charges. Inter. J. Nanomed..

[18] Li M, Lin YC, Wu CC, Liu HS (2005). Enhancing the efficiency of a PCR using gold nanoparticles. Nucleic Acids Res..

[19] Sang F, Yang Y, Lin Y, Zhang Z (2014). A hot start alternative for high-fidelity DNA polymerase amplification mediated by quantum dots. Acta Biochim. Biophys. Sin..

[20] Zhou X, Shah DH, Konkel ME, Call DR (2008). Type III secretion system 1 genes in *Vibrio parahaemolyticus* are positively regulated by *ExsA* and negatively regulated by. ExsD. Mol. Microbiol..

[21] Shen WH, Hohn B (1992). DMSO improves PCR amplification of DNA with complex secondary structure. Trends Genet..

[22] Fredricks DN, Relman DA (1998). Improved Amplification of Microbial DNA from Blood Cultures by Removal of the PCR Inhibitor Sodium Polyanetholesulfonate. J. Clin. Microbiol..

[23] Lee CL, Huang HC, Chiu SY, Lee YS, Pan TM (1995). Latex agglutination test for detection of tetanus antitoxins. Chinese J. Microbiol. Immuno..

[24] Siebert PD, Larrick JW (1992). Competitive PCR. Nature.

[25] Denman, A. M. *Molecular Cloning: a Laboratory Manual (3-Volume Set)*. CSH. Vol. 1, Chap 1, 32–34 (2001).

